# Digital Twinning for 20th Century Concrete Heritage: HBIM Cognitive Model for Torino Esposizioni Halls

**DOI:** 10.3390/s23104791

**Published:** 2023-05-16

**Authors:** Antonia Spanò, Giacomo Patrucco, Giulia Sammartano, Stefano Perri, Marco Avena, Edoardo Fillia, Stefano Milan

**Affiliations:** LabG4CH—Laboratory of Geomatics for Cultural Heritage, DAD—Department of Architecture and Design, Politecnico di Torino, Viale Mattioli 39, 10125 Torino, Italy; giacomo.patrucco@polito.it (G.P.); giulia.sammartano@polito.it (G.S.); stefano.perri@polito.it (S.P.); marco.avena@polito.it (M.A.); edoardo.fillia@polito.it (E.F.);

**Keywords:** HBIM, digital twin, multi-temporal 3D models, IFC, metadata, 20th century heritage, rapid mapping, MMS, SLAM, accuracy evaluation

## Abstract

In the wide scenario of heritage documentation and conservation, the multi-scale nature of digital models is able to twin the real object, as well as to store information and record investigation results, in order to detect and analyse deformation and materials deterioration, especially from a structural point of view. The contribution proposes an integrated approach for the generation of an n-D enriched model, also called a digital twin, able to support the interdisciplinary investigation process conducted on the site and following the processing of the collected data. Particularly for 20th Century concrete heritage, an integrated approach is required in order to adapt the more consolidated approaches to a new conception of the spaces, where structure and architecture are often coincident. The research plans to present the documentation process for the halls of Torino Esposizioni (Turin, Italy), built in the mid-twentieth century and designed by Pier Luigi Nervi. The HBIM paradigm is explored and expanded in order to fulfil the multi-source data requirements and adapt the consolidated reverse modelling processes based on scan-to-BIM solutions. The most relevant contributions of the research reside in the study of the chances of using and adapting the characteristics of the IFC (Industry Foundation Classes) standard to the archiving needs of the diagnostic investigations results so that the digital twin model can meet the requirements of replicability in the context of the architectural heritage and interoperability with respect to the subsequent intervention phases envisaged by the conservation plan. Another crucial innovation is a proposal of a scan-to-BIM process improved by an automated approach performed by VPL (Visual Programming Languages) contribution. Finally, an online visualisation tool enables the HBIM cognitive system to be accessible and shareable by stakeholders involved in the general conservation process.

## 1. Introduction

The 3D surveying technologies are largely applied in the framework of heritage conservation plans, and they are focused on reality-based methods exploiting image and range-based techniques to reproduce accurate and dense models of the investigated environments. They are increasingly called upon to deal with information management scenarios and develop ad hoc systems intended to share knowledge about heritage.

The strategies on continuous and rigorous interdisciplinary-based dialogue to find interpretations and knowledge are intended in the broadest sense of the object’s documentation. They are recognised as essential contents in the conservation and enhancement projects, in addition to the consolidated consideration that inspection and monitoring activities should be documented and kept as part of the history of the structure [1].

In this perspective, the architectural heritage and its artefacts, with their historical and social implications, are an engine of cultural development of society for dissemination and outreach purposes, as recommended by the European agenda Horizon 2020 and widely affirmed by the digital transition mission promoted at national and international levels [2]. The scope of the Digitisation of Cultural Heritage embraces communities of researchers who were once less in contact, and who instead, today, are establishing alliances and common purposes by combining scientific-technological points of view, and so-called Humanities fields in a holistic sense. Using this approach, the combination of 3D sensing technologies and reality-based modelling strategies is considered the basis for advanced generative processes. Therefore, the combination of complex geometries and information enrichment in the modelling process can contribute to the creation and implementation of users-sensitive valuable cognitive models.

LiDAR (Light Detection and Ranging) technologies and digital photogrammetry, articulated both in aerial configurations, (mainly from UAV-Uncrewed Aerial Vehicle) and in close-range terrestrial ones, together with the continuous development of mobile acquisition systems (MMS-Mobile Mapping Systems), with the perspective of integration, allows the management of 3D point clouds with various accuracies and densities, adapting them to the needs of both documentation and data sharing [3].

Enriched 3D models, structured with the support of metadata and organised in systems for data management, facilitate the exchange and cooperation between different research sectors [4]. In particular, the potential of Heritage Building Information Modelling (HBIM) environments is an issue of investigating for addressing the requirements of interdisciplinary analyses, with increasing efficiency, in order to guarantee a complete and rich vision of the space, time, and multi-content and multi-scale models required by the complexity and granularity of the architectural heritage [5].

The harmonisation and possibility of interchange of spatial and semantic data are increasingly identified with the Digital Twins paradigm, which evidently takes on specific connotations depending on the purposes for which the systems are designed and in accordance with the singularities of the heritage assets.

This contribution is linked to the project related to the annual grant initiative “Keeping It Modern” promoted by the Getty Foundation, which in 2019 awarded the conservation project developed by researchers from the Politecnico di Torino and other institutions for the Torino Esposizioni complex, conceived and built by Pier Luigi Nervi in the 1950s [6].

In the framework of conservation plans deeply involving structural aspects, the awareness that the precise detection of geometric characteristics of buildings and their structures and parts can provide an important contribution in the diagnosis phase to assess buildings’ health is becoming crucial, and this improves the confidence in sensing techniques and their interaction within the structural interpretation of features [7,8].

**Figure 1:**
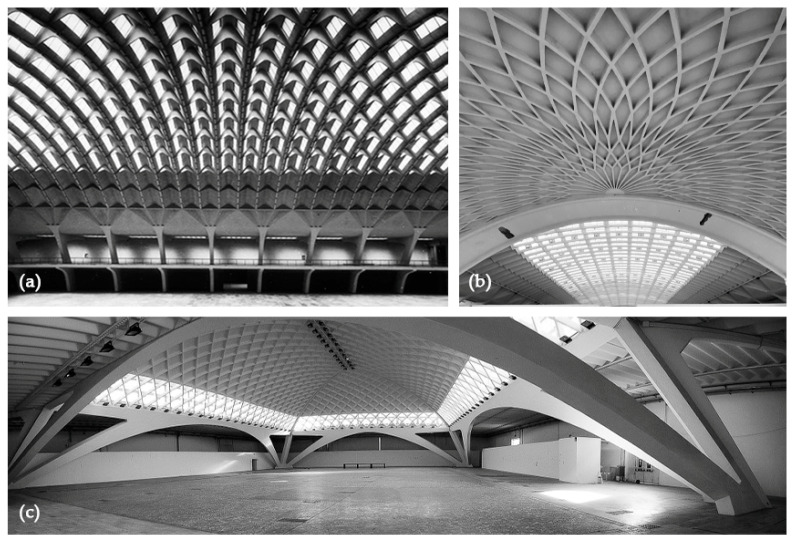
The wide and impressive reinforced concrete vaulted structures of the Nervi’s halls (photos from the archive of the conservation plan—Politecnico di Torino): (**a**) the wide undulated vault form pavillon B; (**b**) the semi-dome ribbed vault of the exedra, pavillon B; (**c**) the inclined arches from C pavillon.

The architectural assets related to the modern movement of the 20th century [9] present completely new spatial conceptions (Figure 1), such as very wide span and thin shell and also, due to their still somewhat limited life, inspired new reflections pertaining the uniqueness of conservation approaches requested [10].

The structure of the paper provides in Section 1.1 a rapid overview of the research fields connected to the HBIM environment, which are currently lively sectors of investigation in the scientific literature. Section 2, concerning the goals, identifies the research motivations of this paper by correlating them to the case study and its challenging aspects; Section 3 presents how data acquisition activities have been made versatile to the user-oriented approach (Section 3.1), and Section 3.2 presents the experimentation of an innovative MMS solution used in interiors. Section 3.3, briefly introducing the consolidated methods of as-built modelling, is directed towards the presentation of a proposal for the use of visual programming language (VPL) to make semi-automatic the conversion of mesh models into BIM objects.

Section 3.4 is intended to clarify the methods of enriching the information of the parameterised model. The final Section 4, results and discussion, and Section 5, conclusions and perspectives—are intended to comment on the results obtained and project them on their adaptability to other case studies and further development prospects.

### 1.1. User-Sensitive HBIM Archives: Beyond Parametric Geometry

The HBIM work environment, which identifies both the Heritage BIM and Historical BIM concepts, involves and connects a series of implications ranging from the geometric organisation of models structured and managed in the parametric form to the archiving of quantitative and qualitative data, which may imply the study and extension of standards, which in BIM corresponds to the IFC, in addition to the complex of different finalisations according to the purpose of conservation, restoration, renovation, rehabilitation, etc., concepts that the Getty thesaurus [11,12] clarifies together with many others.

There are reviews that facilitate the knowledge of an overall panorama of experiences carried out. Many times they are purposed to highlight the connection between the geometry and the structural characterisation of the model elements as well as their spatial, topological and semantic relationships [13]; other times, they highlight the semantic definition of the model offered by the BIM approach by identifying interrelated implications between the functional, organisational, informational and technical ones [14].

More recently, Jouan and Hallot in 2020 [15] propose a model based on the conservation process as defined by ICOMOS, and, again in the field of reviews [16], propose a classification that aims to identify different maturity levels of HBIM experiences with reference to the archiving of photos and other texts and documents, to historical stratification, to previous interventions, to the degradation patterns, to the construction materials with their properties and characterisation, to the predictions of degradation development and to the architectural style and grammar.

There is no shortage of works that focus on the combined use of HBIM with different communication technologies [17] or approaches that aim to use public domain software resources [18]. By limiting the point of view to the areas of research that can be compared with those addressed in this study, thus we go beyond, for example, the GIS/BIM scenario, which is certainly in growing development [19,20]. It is possible to identify that a sector of investigation that arouses great and vital interest are the strategies for the *as-built* geometric modelling deriving the prior sources from reality-based 3D surveying techniques, both laser scanning and photogrammetry, that now presents a dense amount of consolidated solutions (see Section 3.1).

In these premises, the interest in artificial intelligence (AI) techniques is also rapidly growing, which aim to deal with the heavy and time-consuming process of identification and segmentation of unstructured point clouds in a more automatic and productive way, to obtain labelled clouds that identify the different parts of the architectural organism. Many studies in the field of architectural documentation are, in fact, investigating the use of machine and deep learning techniques [21,22,23] to enhance the sustainability of the modelling process, limiting the involvement of human resources in the recognition and segmentation of unstructured 3D clouds that become automatic.

In this sense, incorporating approaches that streamline and automate the data modelling, integration, and management phases has become widespread in the fields of engineering and architectural design, such as visual programming algorithms [24]. Specifically, VPL (Visual Programming Languages) makes extensive use of advanced features to support modelling algorithms, revolutionising the coding processes by replacing traditional written code with visually-based node tools to perform specific and independent functions from main library packages (This process is particularly evident in VPL modelling software such as *Grasshopper*^®^ for Rhinoceros 3D and *Dynamo*^®^ for Revit). These visually-based nodes are inserted into a larger, hierarchical system of parametric rules, making programming more accessible to non-experts.

While VPLs were initially used to generate parametric geometries within CAD3D software for architectural design, the development of BIM processes has broadened their application field [25] for integrating geometric and semantic data into 3D parametric models. Particularly, in geometric object-based reconstruction applications, the need of creating elements of different forms’ complexity (linear, non-linear, round, dynamic, etc.) currently triggers challenges when it comes to geometry construction and documentation across different project stages [26]. Specifically, for this reason, in the case of surfaces and objects of historical heritage that come to facilitate the *scan-to-(H)BIM* workflow more and more extensively.

Furthermore, since HBIM is a sector of study and implementation of information which, like the heritage science sector needs interoperability but upstream of controlled and precise definitions of concepts, the use of ontologies and of semantic information management are rapidly developing [27,28].

## 2. Goals

### 2.1. Digitisation Project for the Conservation Plan of Torino Esposizioni

The conservation project of Torino Esposizioni intends to respond to the expectations of the city of Turin to boost the reuse and reactivation of the admirable vaulted environments designed and built by Pier Luigi Nervi (1947–1950). The conservation project is particularly focused on the aspects of structural and seismic vulnerability [29], as well as representing an exemplary case study to investigate the main implications of the digitisation of heritage [30], as other examples implemented [31].

The reuse of this architectural complex involves the challenge of guaranteeing a new life to the concrete structures conceived and built many decades ago. Some questions were raised about the durability of the materials and the experimental technologies used. In fact, the ferrocement in the Nervi’s conception aims to respond firstly to the need for flexibility and prefabrication, through the design of thin structures that are resistant in shape and, at the same time, and secondly to respond to the cost optimisation required for Nervi [32,33,34] by the client. This has been reported in the literature as “*sistema Nervi*” [35].

Furthermore, this type of construction from the middle of the last century does not meet the current seismic assessment conditions required by recent legislation, which demands restrictive standards for both new and existing buildings. In this perspective, the Torino Esposizioni complex falls fully within the buildings of the 20th century, for which the Madrid-New Delhi Charter provides that in the presence of interventions necessary for environmental conditions or transformations of any kind, the conservation project must guarantee the requisites of “cultural significance, authenticity, and integrity”. These criteria are particularly critical to be respected due to the lack of analysis of the durability of the materials, given the short time perspective with respect to the construction.

**Figure 2:**
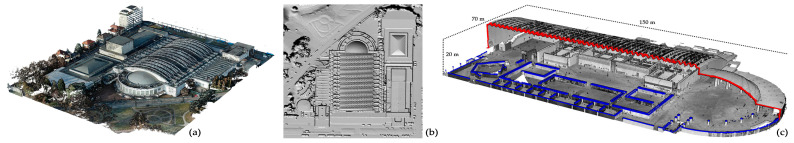
(**a**) Point cloud derived from the photogrammetric process; (**b**) Shaded view of the Digital Surface Model derived from the photogrammetric process; (**c**) Horizontal (blue) and vertical (red) sections of the point cloud (pavilion B) collected using the Swift system.

In particular, a goal of the study we present is to strengthen the suitability of reality-based techniques (photogrammetry and laser scanning) with the aim to obtain accurate and dense detection of complex surfaces that the metric digitisation faces. The 3D survey strategies adopted using such techniques are quite independent of the shape of the structural elements and construction systems, even if materials and the general configurations of the spaces greatly influence the results, making upkeeping of the single reference system and managing huge amounts of data very challenging (Figure 2).

Nevertheless, with regard to the 3D survey using innovative techniques, it is necessary to consider that structural engineering investigations certainly require a high accuracy of the point clouds and 3D models, but at the same time, a balanced density of information and an adequate level of detail [36,37].

In other words, in order to obtain the following results, it must be carefully harmonised:

- a faithful representation of the general configuration of the building;
- a precise geometry of the structural elements;
- guaranteeing multidisciplinary information content.

Finally, as will be referred to in the following paragraphs, one of the aspects to which particular importance was attributed was the possibility of future use of the data: obtaining user-oriented models is a fundamental value for their usability, and it is also necessary to make them compatible with the considerable amount of data in terms of weight of the files. It is in fact important to consider the finalisation of multi-sensor, multi-scale and multi-content models to support different and varied purposes:

- the global knowledge of the spaces by focusing on the thickness of the structural and ferrocement elements, i.e., studying the intrados-extrados problem,
- the characterisation morphology of the structural elements, suitable for the identification of the architectural values,
- the detection of the mechanical deterioration of the elements and the degradation of the surfaces

#### Complex Structures, Subject to Diagnostic Investigations, Challenging Digitisation Processes

The reuse intervention for the Nervi pavilions of Torino Esposizioni has not yet been concluded. Therefore, the multi-temporal 3D metric documentation is not characterised in this case by the classic before-after intervention meaning.

Here the multi-temporal issue is structured on two different aspects: the first consists in having programmatically repeated and enhanced the measurements during the diagnostic investigation campaign carried out by the structural engineering research group [29]. As a consequence, on the basis of the 3D model achieved using Lidar technology, it was possible to merge and integrate other models, often photogrammetric and more accurate, able to document the acquisition position of the data of the diagnostic investigations, and obviously to be able to interpret the results of the investigations with the added-value position in space linked to the surfaces of the structural elements.

A second side of the multi-temporality of digitisation models consists in having recourse to Nervi’s project drawings, which is very often, if not always, a fundamental step in completing and enriching the level of knowledge of the built buildings. Subjecting the archival drawings of Nervi and his school to reconstructive 3D modelling has made it possible to place the designed building and its construction in direct comparison, with the model derived from highlighted reality-based techniques.

As anticipated, the research we present aims to reflect on the methods of managing hybrid and heterogeneous data derived from an interdisciplinary knowledge process that supported the Conservation Plan of the Nervi’s halls. The main purpose, therefore, is to create a 3D digital platform that hosts a digital twin capable of replicating the geometric and semantic contents of the structural elements and their material characterisation by evaluating and exploring the possible solutions offered by the HBIM paradigm. More specifically, it is an issue of harmonising in a single system geometric and radiometric data which characterise the architectural/structural elements, according to a classification that is significant for structural purposes and enriching them with the content of diagnostic tests which together aim at assessing the state of health of the building and anti-seismic characteristics. Even if the multi-sensor techniques offered by the market today are particularly versatile in terms of information density and accuracy and quite adaptable to the infinite variety of materials and configurations of architectural complexes [38], some challenging aspects of digitisation are directly related to the structural conception devised by PL. Nervi for Torino Esposizioni. The complex designed by Nervi completes and enlarges a pre-existing building that already had a role in the city’s intentions, which had promoted its construction, to welcome a new reference centre for exhibition and recreational activities. The first building, damaged during the Second World War, was completely reshaped by Nervi’s interventions: pavilion B (designed in 1948), pavilion C (1950), and the expansion of pavilion B (1954) [39].

**Figure 3:**
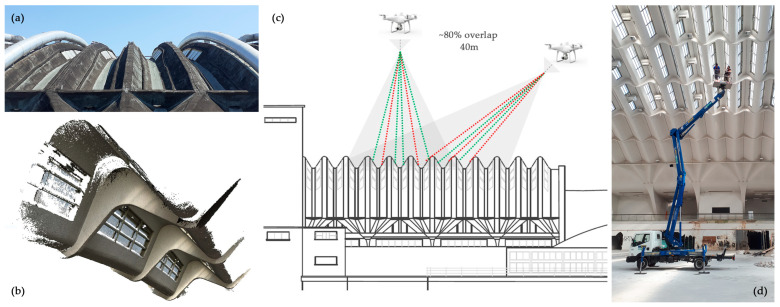
The tailored acquisition strategy for the undulated vault (**a**) made of in-situ precast elements (**b**) is made by integration of UAV (**c**) and close-range photogrammetry (**d**) for the intrados-extrados 3D modelling. The lighting modules and the aeration are elements challenging the 3D surface reconstruction, especially for the ray’s incidence direction (green, good, and red, bad) in photogrammetric matching (**c**).

**Figure 4:**
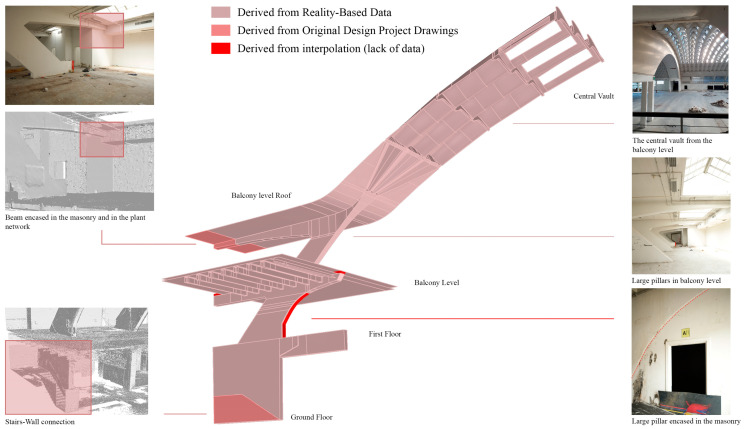
Configuration of the pillar of hall B, in its multi-layered articulation, in underground spaces, the main hall and galleries up to the curved vault. It is visible how the data acquisition for the definition of the real pillar shape was influenced above all by the fact that the pillar was incorporated by the adjacent structures.

Therefore, in addition to the need to cover very large spans suitable for exhibition halls that Nervi challenged with the conception of arches and vaults without intermediate supports, the designer also had to face constraints due to limited resources in a period in which the experimentation of new building materials, such as reinforced concrete whose ductility was discovered, led to the conception of new solutions. The prefabrication on site of the structural elements in reinforced concrete and ferrocement, later patented after the end of the construction, was the new solution proposed by Nervi to save time and resources and obtain structures resistant in shape, with extremely reduced thicknesses, only 4–5 cm for the ferrocement membranes which, therefore, affirmed their malleability, flexibility and lightness. The most challenging aspects related to the 3D data acquisition and clouds modelling phase can be summarised in the following:

- *Impressive and curved reinforced concrete structures.* The parabolic arches of hall C, smaller in size than the overall envelope, cover spans of approximately 40 m × 55 m (distance between the bases of the arches, at the walking surface), while the waved vault of hall B alone has a span of 55 m and it develops for a length of 112 m (total surface in the development in 3D space of 13,280 m^2^). The width of hall B, in the transversal direction, i.e., that relating to the span of the inclined pillars/fan-shaped elements/waved vault system, comes close to 95 m since the development of the inclined pillars in the contrasting direction at the vault is approximately 15.5 m. The half-dome that overlooks the exedra area that faces the river has a diameter of 39 m;
- *Mobile mapping Systems performances in wide and complex spaces.* These incredible dimensions and spans for the time of construction of the building certainly do not worry the ranges of traditional laser scanning, which in the less performing versions easily reach and exceed the 100 m range for determining distances. The immense empty space of hall B instead and the distance of the vault from the walking surface are instead sometimes beyond the limit for handheld scanners based on SLAM (Simultaneous Localisation And Mapping) technology, which was used in the extensive service areas and in the foundation level (minimum height, measured along the vertical, of the element that constitutes the crest of the wave of the undulated vault is equal to 18.30 m from the walking surface). For this reason, hybrid systems have also been tested, based on the combination of traditional laser scanners transported on trolleys and equipped with devices based on SLAM or suitable for recording subsequent scans using ICP (Iterative Closest Points) algorithms, as will be described in the paragraph dedicated to acquisitions using the Swift system of Faro technologies (cf. Section 3.2);
- *The slimness of ferrocement thin shell structures.* In direct connection to the considerations of the previous point, it can be understood that the determination of the thickness of the roofing was a challenging task [29]. Obviously, in this context of intrados/extrados determination, the stiffness of the topographic system and the accuracy of topographic vertices and control points were crucial. While for pavilion C and the dome of the exedra the results were obtained fairly easily, for the waved vault of pavilion B a sample of the wave module was surveyed using a photogrammetric technique with primary shots determined by a forklift. Thus, for that pavilion and its waved vault, it was not simply a matter of a considerable distance from the projection centres of the laser beam, but rather the positioning in the space of the surfaces to be surveyed not in favour of the projecting rays, which made it difficult to obtain the necessary accuracy of the point clouds, as studied [40,41]. The same problem of placing surfaces in space that are not easy to detect was obviously found by the UAV data acquisition in the extrados, as also known [42]. In this case, the quality of the photogrammetric cloud, in addition to being influenced by the incidence radius of the projecting rays, is also influenced by the colour of the surface, which is covered both by a bituminous mantle and by large skylights (Figure 3);
- *3D reconstruction of complex partially-visible objects using topography for accurate reference system.* Another problem that required the extreme accuracy of the topographic coordinates of the control points is the fact that the inclined pillars of hall B (Figure 4), unlike those of pavilion C, despite being visible for most of the bays of the galleries, are incorporated in the walls of the service rooms on the ground floor;
- *Underground environment mapping.* Finally, another type of challenging problem for the use of 3D surveying technologies concerned the underground floor. It is accessed mainly from the curved stairs at the end of the exedra, forcing it to be able to connect the reference system via the topographic network, only from this opening. The existing possibility of performing 3D survey paths with handheld and SLAM-based scanning systems would have benefited from closed paths incorporating traditional terrestrial scans, but such passages were blocked by the compartmentalisation strategies planned for safety reasons.

## 3. Material and Methods

### 3.1. An Object-Oriented Approach for the Integrated 3D Reality-Based Survey

In order to acquire the primary data, it was necessary to plan an extensive 3D metric survey campaign [6,29], allowing the integration of heterogeneous information in a single reference system. The main aim was to exploit the versatility of image and range-based methods to use them individually or in their integration, with approaches to be adapted to the specific purposes required by the different configurations of the architectural complex and its parts. The complete documentation of the Torino Esposizioni halls complex, in perspective linked to the cognitive process that underlies the conservation projects, has been developed with the possibility of carrying out targeted in-depth 3D survey analyses characterised by accuracies, resolutions, levels of detail, and collections of geometric and thematic information of different information richness, higher than the standard used for the general configuration; this comprehensive approach is aimed at improving the diagnosis of the whole and of the different parts of the complex.

Some crucial strategies can be summarised by the following points:

- The reciprocal relationship of the parts of the complex, and the intrados-extrados correlation, has always been detected through topographical measurements of related control points to a rigid topographic network of vertices. The external envelope has been addressed with the integrated use of UAV photogrammetry, traditional laser scanning, and exploiting new MMS solutions;
- Although the structural elements (inclined pillars, arches, undulating vaults) have been the main focus of the conservation project, and consequently for the digitisation one, their highly geometric nature would suggest a leading necessity for range-based methods, but photogrammetry and the consequent radiometric information of higher quality than that derived from laser scanning methods, was fundamental whenever it was necessary to document the anomalies and surface degradations, which obviously could reveal more relevant structural problems. Further, all the metric documentation of the diagnostic investigations that were subject to the multi-temporal 3D surveys already mentioned was mainly based on photogrammetric methods, as reported in Section 3.1.2.;
- The extensive use of the terrestrial laser scanning technique (TLS) has certainly made it possible to document considerable portions of the indoor space of the complex, but it has been widely exploited to build the ground truth necessary to validate the experimentation of the MMS system, (Section 3.2).

Therefore, to achieve these goals, it was essential to materialise and measure a georeferenced topographic network, in the WGS84/UTM 32N reference system, through the use of the GNSS satellite technique (Global Navigation Satellite System) as regards the topographic network vertices outside the building (n. 12), and through the use of the traditional topographical technique that uses the total station as regards the vertices deter-mined inside the complex (n. 13). The accuracy of the coordinates of the vertices of this network, after the least squares compensation, is of the order of magnitude of a few millimetres, therefore, consistent with the pre-established objectives of the documentation project, from a perspective aimed at containing the propagation of error (Table 1).

The significant extension of the buildings of the complex, which occupy a block which is adjacent to the regular grid of the city since it lies on the border of the river park of the largest river of Turin, was subsequently surveyed by photogrammetric techniques with the aid of UAV systems. The use of this strategy has made it possible to acquire, from the aerial perspective, a large amount of data relating to the external envelope, including a theatre, a restaurant, and a building intended for the university. Since the 3D survey using other sensors carried out in the indoor spaces of the pavilions took place within the common reference system, the photogrammetric cloud of the rooftop surface is automatically placed in spatial relation to the point clouds of the inside, allowing to compare valuable information about the characteristics and thicknesses of the coverings. Of course, a set of control points measured with a total station was used to metrically evaluate the results obtained (Table 2).

The interior spaces and vaults of pavilions B and C were surveyed using the techniques:

- Traditional static TLS techniques (using phase shift laser scanners Faro Focus3D X330 and Faro Focus3D S120, by FARO ® Technologies Inc. (Lake Mary, FL, USA), accuracy ±2 mm @ 10 m), which allowed the acquisition of 110 scans and more than 4 billion points (next Section 3.1.1);
- Two different SLAM-based hybrid systems for mobile mapping have been employed. The first one is the hand-held scanner system, the ZEB-Revo RT (from GeoSLAM Ltd., Nottingham, UK), and the second is the trolley scanner, the Swift System (by FARO ® Technologies Inc., Lake Mary, FL, USA) (whose next Section 3.2 is dedicated);
- Terrestrial close-range photogrammetry (next Section 3.1.2).

#### 3.1.1. LiDAR Data Collection: Evidence Data from Static-Mobile Approaches

Inside pavilions B and C, 58 and 44 static scans were performed, respectively. The cloud registration processes, based on ICP and the subsequent roto-translation of the single reference system through control points, have provided results that overall amount to a few millimetres (Table 3).

The ancillary spaces, i.e., the rooms and corridors on the side of the pavilions and the basements, were acquired using the Zeb-Revo RT (from GeoSLAM Ltd., Nottingham, UK), whose algorithms work on closed trajectories acquisitions, and whose accuracy on small and medium spaces has already been tested and validated [43]. In fact, it was possible to acquire accurate point clouds during the movement of an operator, exploiting the use of the SLAM algorithm which allows solving the mapping function estimating the position of the sensor along its trajectory together with inertial-related data and, consequently, to perform a three-dimensional reconstruction of the surrounding space during the motion. The average accuracy is lower than that of traditional terrestrial static systems, but still sufficient for the intended purposes of the survey. The main advantage consists of the possibility of acquiring effective spatial data in an extremely short amount of time, not comparatively to traditional terrestrial scans (Table 4).

Together with the use of a hand-held system, the use of the latest generation MMS Swift system by FARO Technologies mounted on a trolley was tested in both pavilions studied. Section 3.1.2 is dedicated to referring advantages and criticalities of this experimentation, and evaluating the global-local accuracy (halls C), so an overall summary by Table 5 is provided for hall B.

#### 3.1.2. Close-Range Photogrammetry for Multi-Temporal/Multi-Contents Digitisation

**Figure 5:**
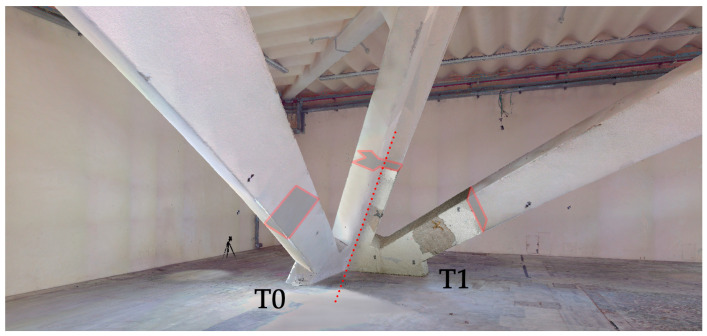
Combined view of the multi-temporal and multi-sensor data on the 3D model (dotted line separation), before and after a number of campaigns for diagnostic investigation. T0: LiDAR point cloud (acquired before the diagnostic investigation campaigns); T1: close-range photogrammetric point cloud (acquired after the first diagnostic investigation campaign).

Regarding the various large and very large-scale investigations that were carried out during the diagnostic investigations acquisitions performed by the structural engineering team, close-range digital photogrammetry was used in both pavilions. As known, digital photogrammetry offers a series of advantages, including its low cost, flexibility, and, above all, the possibility of acquiring higher-resolution radiometric data compared to laser scans, when the lighting conditions during the acquisition are favourable. This approach was used to carry out a series of targeted focuses on the areas and surfaces subject to non-destructive investigations in order to obtain very high-resolution multi-temporal point clouds, characterised by millimetre precision, to spatialise the position of cracks patterns and other non-destructive investigations. Considering the non-optimal lighting conditions, the acquisitions took place with the aid of a tripod, a remote shutter system and two LED panels to ensure adequate artificial lighting of the surfaces of interest. Furthermore, it was possible to acquire some modules of the vault of the pavilion through the use of a lifting basket which made it possible to bring the projection centre of the cameras close to the surveyed surfaces (Table 6, Figure 5).

### 3.2. Exploiting Trolley MMS for Scan-to-Modelling Purposes

The purpose of the experimentation is to validate the Swift MMS point cloud data in different processing configurations, integrating them into the multi-sensor strategy. Specifically, the conducted tests aim at assessing their contribution in alternative to static ones in those cases where the streamlining of automated modelling processes usually requires more efficient workflow, with accurate but optimised and manageable subsampled 3D primary data.

The Swift trolley system by FARO was experimented with in the wide hall spaces, and particularly, it has been tested in Torino Esposizioni hall B and mainly analysed in [44]. The technology is configured as a fusion-based system with three different components working independently: the Lidar scanner (360° horizontal and 270° vertical FOV), the ScanPlan by single laser profile with a horizontal course, the monitoring device (e.g., smartphone) based on a Wi-fi connection and browser GUI. It has been developed for large-extent environments and complex spaces with generally regular floor surfaces to ensure optimal sliding of the trolley on which the double LiDAR system is mounted in a solid system configuration.

**Figure 6:**
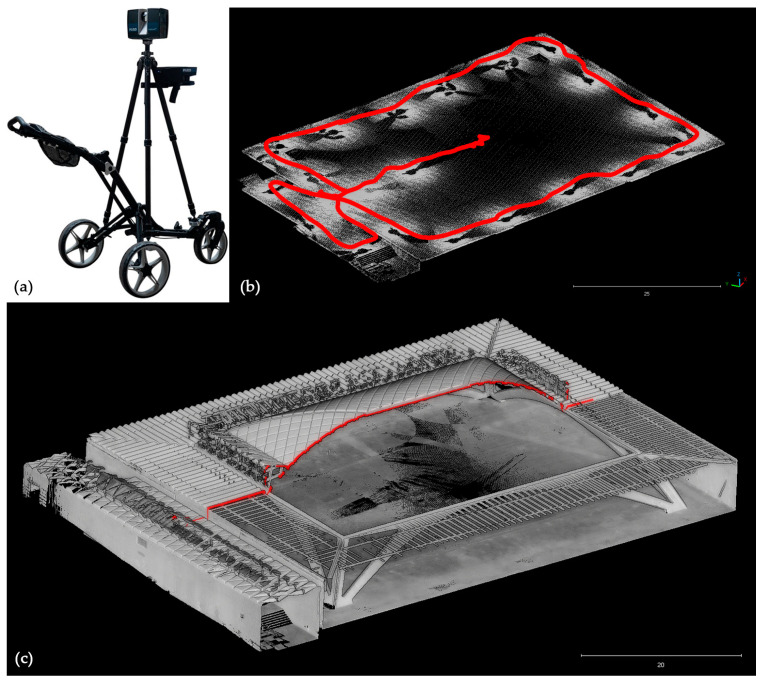
(**a**) FARO Swift system; (**b**) Segmented Swift point cloud evidencing different densities along trajectories in red line; (**c**) 3D vertical section of the point cloud collected with the Swift system (pavilion C).

In the present research case set-up, the core LiDAR sensor capturing the point cloud data and radiometric content is the FARO Focus Plus S series laser scanner with an operative range of up to 350 m. The data acquisition works thanks to an integrated positioning solution based on SLAM algorithms working with the ScanPlan lidar profile, mapping the space in which the system is moving with a maximum range of 20 m. The horizontal profilometer is responsible for the trajectory estimation and preliminary space reconstruction, and generation of a medium-density point cloud (here named *Mobile* point cloud scan) in Figure 6. This is useful for pre-positioning the scan data during the moving acquisition progression. The actual range-based acquisition is based on the combination of two different scans typology: the *long-interval scans* and the *anchor scans* [44]. If the former is represented by the acquisition of the standard static scans (with tens of minutes scanning and RGB captured data), the latest is a faster version of a static scan, performed along the trajectory characterised by short duration and lower densities, according to the setting parameters (without RGB captured data in the present research release but implemented in current system configuration). *Anchor scans* (Figure 6) generally require 15–20 s and are captured each 5–10 m, with a resolution of ¼ (1 pt/mm @ 10 m) and measurements quality of 1× (measured once).

The processing strategy of the Swift datasets can often benefit from integrating a number of static long-interval scans acquired during mobile scanning, while anchor scans constitute the basis for the continuous mobile mapping performance and uniform distribution of denser points. The processing workflow has been recently improved from the first system release. Above all, the system allows taking advantage of anchor scans as both integrated elements of the mobile scans data and a static scans object for all intents and purposes. In the present case, the Swift mobile mapping scan project was developed independently as required by the procedure and later subsequently co-registered with the static scan project acquired in the previous stages of the work (as reported in Section 3.1.1). Starting from the static LiDAR scan registration, ICP average distance errors are approximately 2–3 mm, 71% <4 mm, and 7 mm accuracy on n° 36 CPs targets (dataset I).

The Swift scans processing reported the following accuracy statistics on hall C (earlier test in hall B reported in [44] and synthesised in Table 5):

- ICP registration of the *anchor scans* block: 5 mm accuracy (47% <4 mm) (dataset II).
- ICP registration of (II) with *Mobile* scan 7 mm (dataset III).
- ICP Swift block (III) with the *static* block (I): 11 mm accuracy (22% points <4 mm).

Specific characteristics of this type of data should be underlined in relation to the quality and precision of the point cloud since they are typically path-related and, therefore, distinctive of a mobile system. The point distribution and density are connected with the trajectory. In Figure 6 the floor point cloud with the trajectory, and density distribution according to the scan sensor position during the acquisition is visible. Considering a surface density in 1m^2^ samples it is possible to underline great differences in density distribution, taking into account two floor samples and two vault samples (Table 7 vault areas), in the two types of scans: *anchor scans* (II) and *mobile scan* (III). In dataset (II), floor level density is between 1000 and 8000 pts/m^2^, depending on the sample distance from the trajectory area, against dataset (III), richer in points density, between 10,000 and 140,000 pts/m^2^. In the vault modules statistics, *anchor scans* (II) have 6000 pts/m^2^ for the ribbed vault elevation, and 17,000 pts/m^2^ for the corrugated slab elevation, instead of *mobile scan* (III) where density is exponentially increased, 30,000 pts/m^2^ for the ribbed vault elevation, and 170,000 pts/m^2^ for the corrugated slab elevation.

The precision of the point is characterised by medium levels of planarity and surface noise, especially at long distances, and outlier error points in correspondence of reflectance surfaces. The cloud pattern typically shows cross-profiles due to the combined movement-rotation of the sensors. In some cases, these characteristics emerge as bottlenecks, as already highlighted in [44]. In these cases, the use of the point cloud requires further processing and filtering operation. The validation of the clouds from the point of view of local and global metric accuracy has been addressed in [44], while here, the goal is to validate their empirical use in *scan-to-BIM* modelling processes.

#### Point Clouds Performance and Accuracy Validation on As-Built Modelling: Selected Samples

In the surface samples analysed, the points features of the Swift system with *mobile scans* data and *anchors scan* data have been compared to the static LiDAR ground truth.

The workflow analysed and applied to the primary dataset is the points-to-mesh-to-NURBS pipeline (see Section 3.3), highlighting that for each sample, the same configuration of plans was used for the extraction of the profiles, and therefore, the reconstruction of the NURBS, to make the models comparable, and therefore, the evaluation effective. The ultimate NURBS surface model generated from *Mobile* and *Anchors* is finally compared with the LiDAR point cloud ground truth.

Considering the tolerance value of ±5 mm error between the generated surface and the reference ground truth LiDAR data, as visible in columns B and D, Table 7, the NURBS generated from *mobile scans* point cloud (column A) holds for each structure type the highest percentage of deviation errors < 5 mm, in comparison to the NURBS, generated from *anchor scans* point cloud (column C).

- 76.3% vs. 57.4%, for the inclined pillar segment;
- 51.8% vs. 36.8% for the ribbed vault module;
- 90.5% vs. 72.8% for the perimetral corrugated slab portion.

### 3.3. 3D modelling: From Unstructured Data to Object-Oriented Models

The modelling phase, in this case, is oriented towards four main objectives, which, however, are not independent, but all contribute to generating the cognitive HBIM model which must be enriched with diagnostic information (Section 3.4) [29].

1. The generation of accurate reality-based geometries of the complex reinforced concrete architectural-structural elements based on point-clouds data;
2. creation of a 3D object from these geometries, capable to be integrated into a topologically correct model consistent with the real structure configuration;
3. definition of 3D objects which can be imported and integrated into an (H)BIM-space modelling, initially as a Metric Generic Model and then converted into specific Structural Families;
4. 3D objects modelled outside a parametric space could be recognised by BIM Revit^®^ space as host objects to operate actions for different levels of customisation.

The adoption of generative processes tailored for such complexity of structural elements can be faced with the so-called “reverse modelling” approach. Generally, as in these cases, the starting point is reality-based data, usually 3D optimised point clouds from image- or range-based techniques, we consider an *as-built* approach for surface approximation, unlike surface- or object-based modelling from scratch in CAD/BIM environments [45].

In fact, the procedure of surface generation created from reality-based data can be summarised in literature as a surface generation with point-based and profile-based approaches, in the sense of the use of points interpolation or profiles interpolation, derived from generative geometries. In scan-to-HBIM approaches, they have been defined as GOG 9–10 [46]. The implemented workflow based on point cloud segmentation, according to elements complexity, and semi-automatic surfaces recognition have been conducted according to the analysis of morphologies for different structural elements (figure Section 4.2): planar surfaces as floors and walls, exedra wall, SAP (reinforced concrete-brick) barrel vault, waved and parabolic vault, ribbed semi-dome apse, perimeter slabs, inclined pillars, hall C arches.

**Figure 7:**
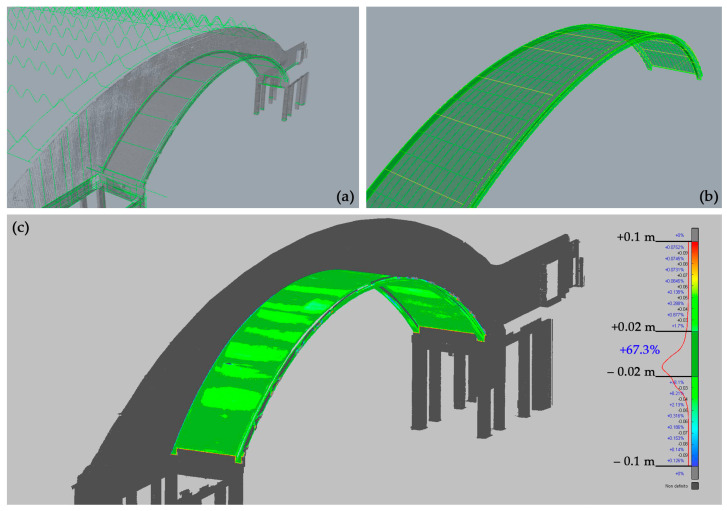
(**a**) Profiles extracted from the reality-based mesh; (**b**) Interpolated NURBS surface; (**c**) Discrepancy analysis between the modelled NURBS surface and primary data.

**Figure 8:**
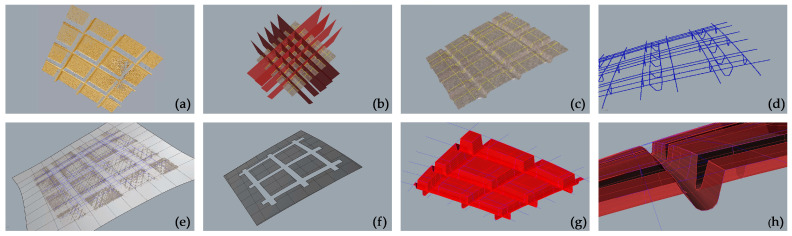
(**a**) Segmented point cloud; (**b**) 3D mesh (computed from the point cloud) with the planes for the automatic extraction of the significant profiles; (**c**) 3D mesh and automatically extracted profiles; (**d**) Geometrised profiles; (**e**) NURBS surface interpolated from the sections; (**f**) 3D modelling phase; (**g**) Final NURBS model; (**h**) Detail of the final NURBS model.

The different generative modelling strategies for the points-to-NURBS object generation are declined according to the starting surface, (A) based on a plane or identifiable geometric primitives interpolation procedures, and (B) based on more complex geometric primitives to be analysed using profile extraction.

(A) Planar and planar-like surfaces extraction:
  - by plane interpolation, as the exedra walls;
  - by surfaces interpolation from generative primitives and curves profiles extraction as the SAP arch in Figure 7;
(B) Complex surface extraction is based on profile extraction and curve interpolation.
  - The use of cutting planes is strategically located profiles primitives extraction with cutting planes and profile curves connection with NURBS modelling (Figure 8).

#### 3.3.1. *As-Built* Modelling from Point Cloud to NURBS toward HBIM-Fitting Object Enrichment: A Novel Approach to Automation

Considering the ever-increasing direction of automation processes in scan-to-BIM processes, as introduced, it was proposed in this research to test the potential of VPLs to develop a semi-automatic modelling process for various structural elements. The process works from NURBS to the BIM environment in order to generate a semantically characterised object in a structural category so that it becomes an interoperable object capable to host, for example, structure elements parameters and reinforcement rebars (See Section 4.3).

**Figure 9:**
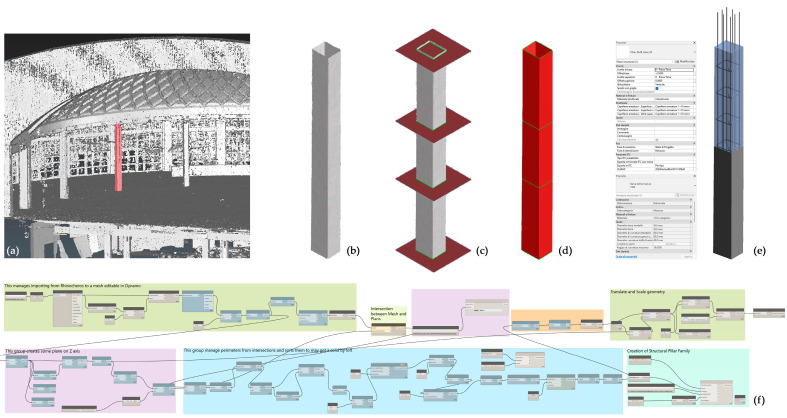
(**a**) Three-dimensional point cloud of the apse (the considered pillar is evidenced in red); (**b**) 3D mesh of the pillar; (**c**) 3D sections for the extraction of the significant profiles; (**d**) Generation of the NURBS surface from the extracted profiles (**e**) BIM object with the related properties table; (**f**) Diagram of the VPL script from Dynamo (See Figure A1)).

In particular, pillars were selected in two different complexities as case studies within the project, one to represent a linear-shaped pillar (Figure 9) and one with an irregular shape (see Section 4.3 and related figure). These elements, derived from the Swift *mobile* point cloud data, were initially processed in a preliminary mesh model, and then imported into the BIM software by VPL. Specifically, Rhinoceros 3D software was used to generate and edit the mesh of these elements, while VPL modelling software such as Grasshopper for Rhinoceros 3D and Dynamo for Revit was used to automate the process of recognising/modelling within the BIM environment. By assigning a specific Revit System Family (*Structural Pillars*) to the generated elements and considering the linear-shaped pillar, it was possible to create a semantically enriched BIM object, geometrically not parameterizable but with a unique ID, and also assign specific host element properties to these objects. This feature allowed for the inclusion of loadable families within the element-Host element and enriched the model with additional geometric and semantic information. In this specific case, an iron reinforcement was inserted inside the generated structural pillars, and according to reality-based data (See Section 4.3 and related figure).

The procedural Dynamo workflow (Figure 9, see also Appendix A), can be summarised as follows:

- Step 1: Import the Rhino model (mesh) into Dynamo. Once the model (*.3dm) is imported into Dynamo, the primary objective is to generate a new dictionary, which is a data type consisting of a collection of key-value pairs. This makes it possible to properly read the imported file; then it is possible to extract the vertices from which it is composed in order to join them later to generate surfaces useful for creating a mesh that can be decoded by the program.
- Step 2: Generation of n Section planes normal to the imported model. In this specific case, to make the process more standardised, a specific script was created to automatically generate a series of planes starting from an input parameter (plane offset, according to the object complexity).
- Step 3: Intersection of Section Planes with Mesh (for profiles extrapolation). Once the section plans were generated, by intersecting them with the mesh, it was possible to proceed with the extrapolation of profiles. In order to do this, two specific customised nodes were used, Sastrugi-Sort points as perimeters and Remake Polycurve, created by Ewan Opie (see Acknowledgements).
- Step 4: Creating a solid by loft assigning translation/scale to the model. By using the planar profiles extrapolation, a solid was created through a loft between the various polylines, thanks to the Solid.byLoft node. However, before importing the generated model into the Revit environment, it is needed to scale it according to a known dimension and then translate it to the origin of the Dynamo workspace.
- Step 5: Creating a new Revit Family and application of the iron reinforcement. The newly generated geometry was used to create a specific *Structural Family*. The noteworthy aspect of this family is its capability to act as a Host Family, as it can host other elements within it, such as all of the components that belong to the metal structure.

### 3.4. Informative Enrichment of HBIM Model

As anticipated, to ensure that HBIM of the Turin exhibition halls functions as a smart archive of information concerning the state of health of the structural elements, and so that the results of the diagnostic investigations can constitute a wealth of richer knowledge thanks to the reciprocal spatial relationship and interconnected with the geometric model, their nature, the format of the data, and the spatial relationship with the corresponding geometric elements were examined.

The diagnostic project implemented by the team of structural engineers [29] was truly vast and the first analysis of the types of results led to clarifying the elements that can host the results of diagnostic investigations (Table 8) and the types of data to be archived, presented in the following table (Table 9). Since the nature and format of the information is less variable than its content and meaning, some results of the diagnostic tests, chosen as samples, will be taken into account and archived, addressing relations to the structural elements models of hall C subject to the test.

In this specific study, which can be considered typical of cultural heritage research, the need to store reliable information regarding the diagnostic test performed and the resulting outcomes can be coupled with the investigation of the potential and critical issues regarding the generation of an IFC model as an HBIM database (see Section 3.4.1).

Considering Table 9, it must be highlighted that the IFC standard, and therefore, Revit, uses the term column to identify a vertical structural element, which generally functions as a support for beams, arches, floors and vaults; in this context, it should be understood as belonging to IfcStructuralElementsDomain.

#### 3.4.1. Information Structuring according to the IFC Model

With the goal of making an open and efficiently usable HBIM archive, it is proper to draw attention to the IFC format. As well-known, the IFC format is the open-source standard used for the exchange of BIM models, developed by BuildingSMART, which has become an open format regularised by the International Organisation for Standardisation (ISO). Currently, the IFC formats and standards are constantly being developed and updated to adapt to the needs of the AEC industry. Hand in hand, IFC standards are updated in ISO 16739-1:2018 [47] and ISO/TC 184/SC 4 [48].

The IFC4.0.2.1 [49] is currently the most supported and stable edition, conveying 3D property and geometry data, ensuring a better round trip of the exported models. Therefore, the concept of Model Reference View (MVD) assumes a fundamental value in the IFC schema, since it is used to identify data exchange requirements for software applications and their interoperability.

**Figure 10:**
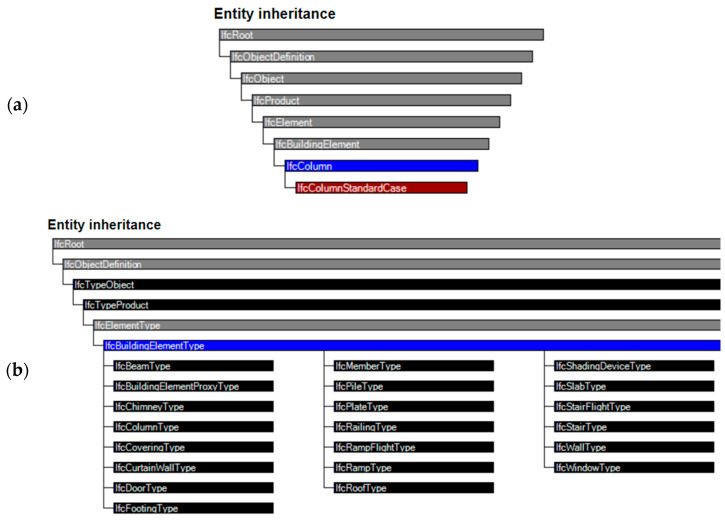
IFC entities classification and their inheritances: (**a**) The entities inheritance focused on specific standard elements; (**b**) The inheritance focused on different building element types (BuildingSMART).

IFC properties can be fully formulated and stored in the IFC definition data model. Some BIM applications such as Revit can automatically assign internal properties and then instances to default properties that are compatible with the IFC standard. This ensures that the information related to an object model is provided and interoperability is ensured. Figure 10 refers firstly to the hierarchic relationship among IFC entities till IFCcolumnStandardCase (with the meaning of a generic vertical structural element); then the inheritance relationship among building entity types (BuildingSMART). The concept template Property Sets (Pset) describes how an object occurrence can be connected to single or multiple property sets.

## 4. Results and Discussions

The 3D survey and modelling enabled to obtain three kinds of results in three different directions: the ability to analyse the shape, anomalies and decay of the building parts and structural elements (Section 4.1), the structured models organised according to the organisation of elements significant from the structural point of view (Section 4.2), the enrichment of the HBIM model (Section 4.3) and visualisation and sharing (Section 4.4).

### 4.1. Shape, Anomalies and Decay Analyses

The analysis of 3D survey products offered the chance to investigate and represent very typical decay and materials deterioration, according to the information acquired on the basis of the image or range-based method:

- Crack extensive patterns in the ceiling of the basement floor, surveyed by lidar and photogrammetric reality-based technique.
- Large depressions of the horizontal surfaces of the roofs were detected by the DSM derived from UAV photogrammetry.
- Efflorescence from humidity and micro-cracks on the surface of the pillars and arches mainly derived from very large-scale photogrammetric applications and related orthophotos
- Evaluation of the deviation from the project generating curves, in general for all the arches and vaults, and in particular for the SAP vault of pavilion B, for which a lowering in correspondence with the semi-dome was interpreted not as a yielding, but as a need of connection between two surfaces implemented in the construction phase matter of which Nervi must have been aware.

Other evaluations of whole project shapes and anomalies conceived by Nervi are less typical, so some main analyses and resultant considerations are referred to as follows:

- *Assessment of the parabolic nature of the generative curves at the base of vaults and arches*.

Many authors agree that the “reckless static intuitions” of Pier Luigi Nervi are combined with a structural conception that is inspired and ends in geometry, from which the designer draws an aesthetic and constructive synthesis [50]. The study of geometric primitives or generative curves is one of the fundamental steps of the as-built strategy; at the same time, this identification can have the role of hypothesising or assessing the nature of any shape anomalies, as it is possible to verify the deviation between the accurate reality-based models and the generative geometries. This investigation was adopted for the surfaces of the vaults and for the arches of the two halls, which was followed by the assessment that Nervi had conceived parabolic curves, tracing them by points as evident from the drawings kept in the archive.

**Figure 11:**
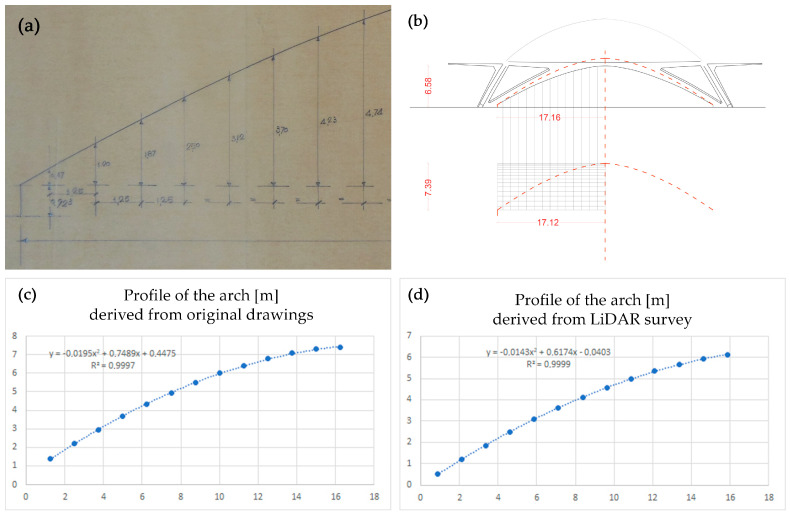
Comparison between designed parabolic curves (**a**) and the real built arch surveyed in (**b**), where the red dash line represents the projection of the designed one. On the bottom, the assessment of the parabolic nature of the two curves and their equations in (**c**) the designed one and in (**d**) the achieved one. R^2^ quality parameter of the regression model is close to 99% in both cases.

We present an example concerning the arches of pavilion C as they are the most complex, given that they present a double curvature, both in vertical and horizontal projection, a sign that they were conceived in space to counteract the weight of the vault which certainly has non-vertical thrust loads. The analytical verification took place with the use of Excel software which, on the basis of the spatial distribution of the points, provides the equation of the parabolas conceived by Nervi and the one derived from the LiDAR survey (Figure 11); moreover, the R^2^ parameter, also known as the coefficient of determination, statistically measures the quality of the regression model in adapting to the observed data. The following figure summarises that the curves traced by Nervi are certainly parabolas (R^2^ 99%), that the construction of the arches represented by the LiDAR models follow a parabolic trend perfectly (R^2^ 99%), in both projections and that, however, does not correspond to the preserved drawing, a sign that Nervi conceived the curves in several moments and not all the documentation is kept in the archive [29,51].

- *Deviation among conceived/designed and built shapes of arches and vaults.*

**Figure 12:**
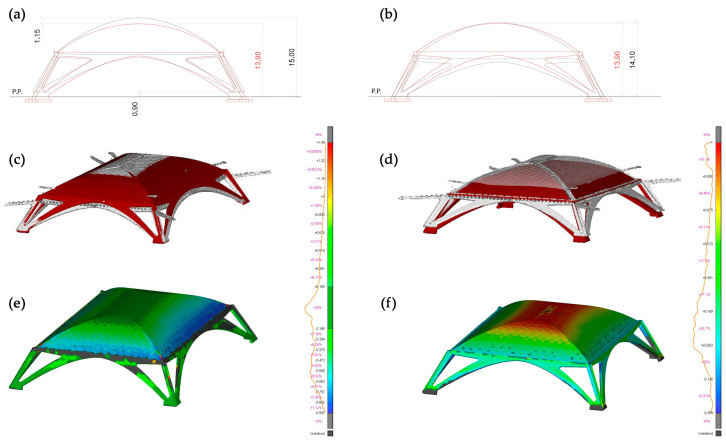
Comparison between LiDAR model (black in (**a**,**b**) and grey in (**c**,**d**) and a model virtually generated starting from Nervi’s drawings (red in (**a**,**b**) and dark red in (**c**,**d**)), in 2D projections and using ICP surface comparison (**e**,**f**). in the first column (**a**,**c**,**e**) the alignment based on floor level, in the second column (**b**,**d**,**f**) the alignment is based on impost vault level.

The inspection of the differences between designed and built parabolas suggested continuing the comparison on other dimensional and geometry aspects of arches and vault shapes. Surprisingly, it was found that the archival drawings known and studied so far must certainly not coincide with the final project which, therefore, evidently developed according to a slow process of approaching the final balance and harmony achieved. The comparison took place both by comparing the projections of arches and vaults and by again employing the ICP algorithms for a 3D spatial comparison between the LiDAR model and a model virtually generated starting from Nervi’s drawings, Figure 12 [29].

### 4.2. The Structural Element-Based NURBS Modelling

The as-built workflow, based on different strategies, and largely founded on profile extraction via sequences of sections, with the aim to model the surfaces of interest, enabled the optimisation of the final 3D models of structural elements and building parts without degrading the metric accuracy. The derived accurate NURBS surfaces many times present millimetre-level deviations from the original physical model and in case of major complexity very few centimetres.

**Figure 13:**
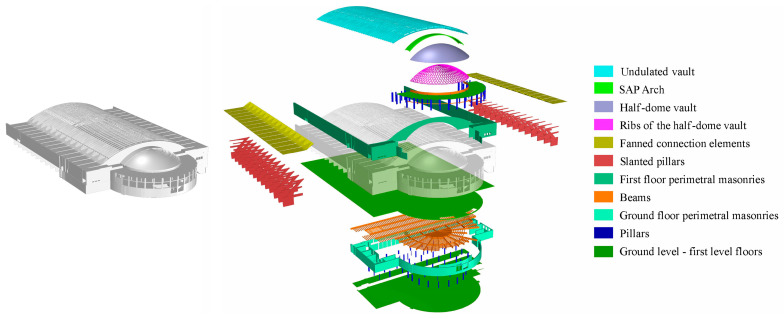
The extremely articulated NURBS model of hall B including both structural elements typical of the architectural and structural conception of Pier Luigi Nervi and standard parts of the building.

Figure 13 shows the overall model created for pavilion B, which includes both a classification of standard building parts, such as floor plans, slabs, stairs, standard-shaped pillars, vertical walls and partitions, as well as extraordinarily original structural elements conceived by Pier Luigi Nervi, from the inclined pillars of hall B, to the waved vault, to the fan-shaped structural elements, to the ribbed semi-dome of the apse [29].

### 4.3. The 3D Archive Implementation: Decay and Diagnostic Information Mapping

**Figure 14:**
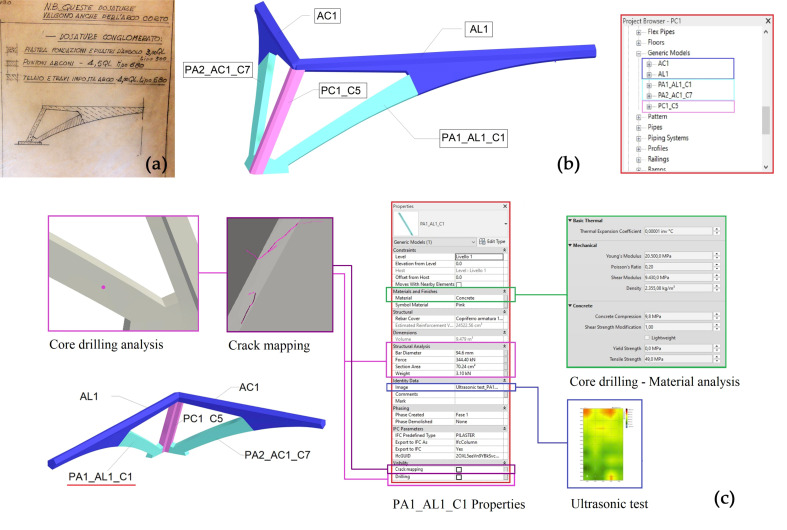
The family properties related to corner combination of pillar/arches elements in hall C. (**a**) different conglomerated blend according to a Nervi scheme. (**b**) different codes assigned to the analysed element; (**c**) objects properties and diagnostic investigation information implemented in relation to geometric model.

The last step of this study, pertaining to the 3D archive implementation by diagnostic investigation result information, has been applied to a representative portion of the structure of hall C, the combined system formed by the corner pillar (coded as PC1) and the large arches segmented elements (coded as Pa1_Al1_C1 and Pa2_Ac1_C7), cast separately by Nervi because of the different conglomerated blend (upper part of Figure 14).

These arched elements, which present a double curvature in space, as mentioned with a parabolic trend both in vertical and horizontal projection, and which vary their resistant section along their development, certainly cannot be transformed into parametric BIM models. In other words, they are implemented in Revit as models of generic families, coded according to the classification adopted by the conservation project and subsequently assigned to a specific entity of structural domain, the structural pillar family.

**Figure 15:**
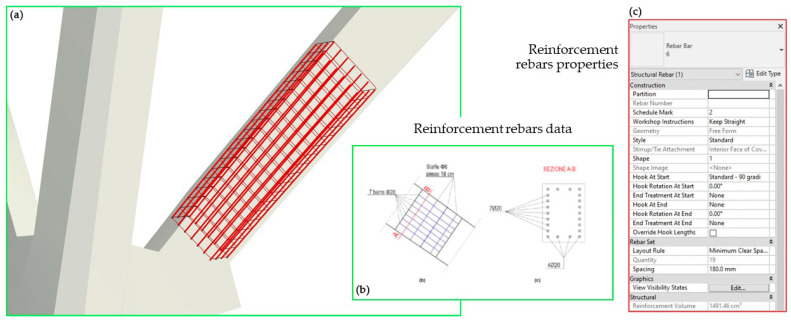
The inclined pillar object in the viewer (**a**), the reinforcement rebars’ parametric modelling (**b**) and related properties (**c**). A–B section in (**b**) show the reinforcement configuration.

Each family has been filled in with information regarding diagnostic analyses and the resulting outcomes, as reported in the following bullet list:

- *Crack mapping.* Cracks were modelled in Rhino contextually to the structural element on which they are located by extruding along a set length. They were then imported separately into the same family and characterised by a “Yes/No” visibility parameter so that they could be visualised as needed.
- *Cores.* Cores were modelled as buttons and treated with the same logic as cracks; the alphanumeric data attached to them were entered as default properties. Some BIM applications, including Revit, can automatically assign internal properties to default properties compatible with the IFC standard. Not all the information that needs to be entered, however, matches Revit’s default parameters, and not all of them match IFC properties.
- *Ultrasonic testing.* The results of ultrasonic testing are numeric data (speed m/s) in addition to raster images showing the data interpolation on the scan area; the link to these raster images has been included in the element parameters.
- *Pacometric tests*. The results of the pacometric tests lighten the features of the reinforcement present in the various structural elements. Such data were used for automatic modelling of the iron reinforcement within the element, which can, therefore, be considered “as built” in turn. The reinforcement is a parametric element that requires a host (Figure 15). It can be created in Revit directly from the geometry shape by selecting the edges defining the surface or the path along which the bars are distributed. This process can be made automatic with the use of Dynamo, as seen in Section 3.3.1.

### 4.4. Accessing 3D Archive Information

**Figure 16:**
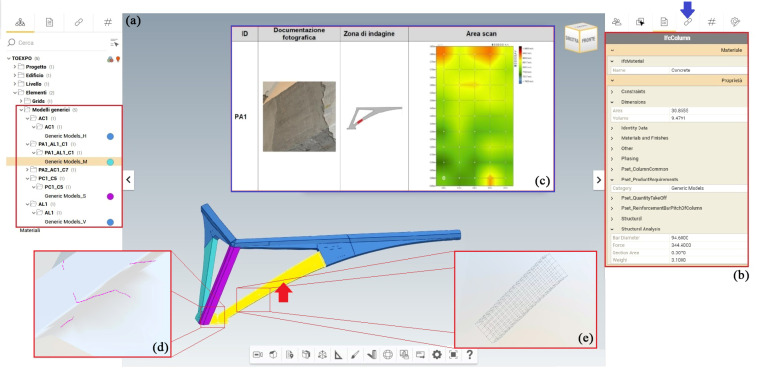
Cognitive 3D HBIM model enriched by diagnostic investigation results navigated by using an open-source IFC viewer. The inclined pillar object in the viewer (**a**), pointed with red arrow in the figure, and the related information n the IFC schema information in drop down menu (**b**). (**c**,**d**,**e**) are visualization data related to diagnostic investigation and reinforcement (Figure 15).

Exporting the HBIM model to IFC2x3 or IFC4.0.2.1 allows interactive visualisation using an open-source IFC viewer. The viewer (Figure 16) allows the exported model navigation in typical modes as displaying elements in a drop-down menu, hiding them, orbiting them, and viewing their properties. In addition, the IFC model can be used by other software, inheriting the current parametric features and the data archived with reference to codes and classifications. For our sample, not all Revit properties and instances match IFC properties; when they do not match, they will be exported in IFC format with the specifications identified by Revit. However, the characteristics of the materials fully match the IFC properties, so they will be automatically exported.

## 5. Conclusions and Future Perspectives

The as-built modelling and information archiving strategies implemented for the Torino Esposizioni HBIM cognitive model can be considered thoroughly in line with the needs of the conservation project. In particular, numerous results of diagnostic investigations and analyses conducted, as well as the patterns and trends of cracking systems or other material degradations, can take on different meanings and provide more complete knowledge about the state of health of the building. They can be examined in accordance with their distribution in relation to the structural elements represented by their digital twins. Indeed, the entire process of generating the cognitive model by adopting the HBIM strategy continues to prove cumbersome and very time-consuming, as the parametric nature is not applicable to the construction, structural and architectural elements in general characterised by non-standardizable geometries; many studies have highlighted, in general, the context of built and historical heritage. The impossibility of applying surface textures using raster maps that continuously represent phenomena whose spatial distribution can play an important role for the purpose of consulting and interpreting the phenomena taking place on the structural elements continues to be a bottleneck. If we retrace the process from the initial point, we can undoubtedly say that the evolution of rapid mapping technologies based on the different SLAM technologies and visual odometry, always coupled with the ICP algorithms that characterise MMS, are to be taken into high consideration as an alternative and integration of consolidated methods. It is prominent that the VPL tools are boosting and can be considered more than promising considering their great versatility and manageability for automating the creation of HBIM models, just as the scenario of AI techniques pushes the process towards automation of segmentation and classification of unstructured point clouds. Finally, as in other fields of investigation, the use of archiving strategies that make use of standards such as IFC in the BIM environment is certainly a starting point for guaranteeing the necessary interoperability, also and above all, this digital twinning sector applied to the field of structural study subsequently proceeds to the use of models for static and dynamic simulations. The experience conducted has shown that it is possible for completely unusual structural elements and architectural parts such as those designed by PL Nervi, to refer to classes of elements consistent with the IFC standard and their classification and coding. This demonstrates that this strategy, which in perspective could be better directed by appropriate facilities offered by BIM software, can become strategic in the context of cultural heritage.

We can certainly state that the approach adopted puts the spatial or non-spatial data at the centre of interest. Data must be coherent, harmonised, in a reciprocal relationship between geometric and semantic ones, possibly archived with the use of shared standards; only in this way can the resulting user-oriented information system be considered an important tool to base the future conservation project of the architectural asset.

## Figures and Tables

**Table 1:** RMSE accuracy for the topographic coordinates of the external and internal vertexes of the architectural complex.

	RMSE Plan [m]	RMSE Elevation [m]
Outdoor topographic vertices	0.005	0.004
Indoor topographic vertices	0.007	0.003

**Table 2:** Metric accuracy of the UAV photogrammetric 3D mapping observed on GCPs and CPs.

	RMSE X [m]	RMSE Y [m]	RMSE Z [m]	RMSE XYZ [m]
GCPs (39)	0.017	0.014	0.009	0.024
CPs (25)	0.021	0.016	0.013	0.030

**Table 3:** Features and statistics of the LiDAR acquisition and processing quality for ICP- and target-based co-registration.

	N° Scans	Employed Scanner	System Precision	Expected Density	Point Cloud Dimension	ICP Registration Accuracy	CPs Target Registration Accuracy
LiDAR scans	58 (B) + 44 (C) + 8 (corridors)	Faro Focus^3D^ X 330 Faro Focus^3D^ S 120	2 mm @ 10 m	>100.000 pt/m^2^	4 mln points	2–3 mm	5–7 mm

**Table 4:** Features and statistics of the SLAM Zeb Revo Geoslam acquisition datasets.

	N° Scans	System Precision	Average Scan Time	Point Cloud Dimension
ZEB REVO scans	8 (ground floor, north and south side) + 5 (underground)	2–4 cm (local, without drift errors)	10–20 min	300 mls points

**Table 5:** Main characteristics of the clouds experimenting the FARO Technologies SWIFT.

	N° Scans/Dimension	Average Path Length	Average Scan Time	Data Accuracy	Point Cloud Density
Swift by FARO Technologies	3 (hall B); 600–700 mln points/scan	400 m	15–20 min	<1 cm @ <100 m 4–5 cm @ >100 m	53.000 pt/m^2^ a terra 23.000 pt/m^2^ rooftop (@20m from sensor)

**Table 6:** Main characteristics of close-range photogrammetric surveys using the camera Canon EOS 5DSR/Zeiss ZE/ZF.2 Distagon T* 25mm f/2.

	N° Images	GSD	Average Shooting Distance	RMSE (GCPs/CPs)
Hall C vault	1045 images	3 mm	13.2 m	9 mm/8 mm
Hall C pillar basement	80–90 per element	<1 mm	3–4 m	about 3 mm

**Table 7:** (**A**) Portion of the point cloud acquired with the Swift system (SLAM-based mobile mode); (**B**) Discrepancy analysis between the NURBS modelled from the Swift point cloud and the LiDAR point cloud (ground truth); (**C**) Portion of the point cloud acquired with the Swift system (anchor scans); (**D**) Discrepancy analysis between the NURBS modelled from the anchor scans point cloud and the LiDAR point cloud (ground truth).

	A	B	C	D
Arch strut	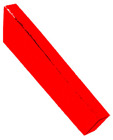	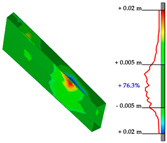	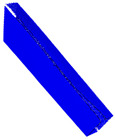	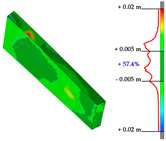
Ribbed vault	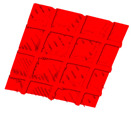	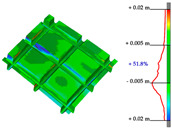	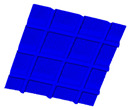	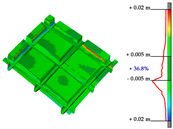
Corrugated slab	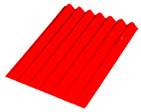	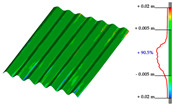	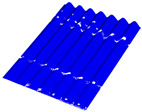	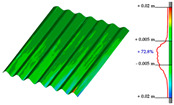

**Table 8:** Structural element types identified in pavilion B, represented by a reality-based cloud (right) and classified by the conservation project (codes referred in the second column).

Element	ID Code	
Shorter Arch	AC_x8	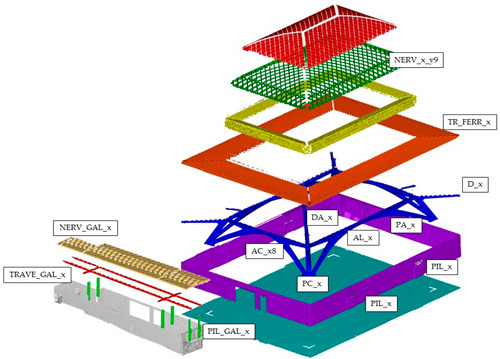
Longer Arch	AL_x	
Central strut of the arches	PC_x	
Strut of the arches	PA_x	
Perimetral pillars	PIL_x	
Diagonal beams	D_x	
Diagonal beam at vault level	DA_x	
Ribs	NERV_x_y9	
Ferrocement beam	TR_FERR_x	
Beams of the gallery	TRAVE_GAL_x	
Ribs of the gallery	NERV_GAL_x	
Pillars of the gallery	PIL_GAL_x	

**Table 9:** Diagnostic analysis data related to specific Revit families and elements.

Type of Diagnostic Test	Type of Results Data	Object to Be Connected
Determination of the sclerometer index	Numeric data	Metric Generic Model/Structural entity
Ultrasonic tests	Raster data	Surface area on the object
	Numeric data	Metric Generic Model/Structural entity
Internal temperature and humidity monitoring	Numeric data	Metric Generic Model/Structural entity
Coring	Numeric data	Metric Generic Model/Structural entity
Determination of carbonation depth	Numeric data	Metric Generic Model/Structural entity
Compression tests on extracted concrete samples	Numeric data	Metric Generic Model/Structural Columns
Tests for the determination of the elastic modulus on the extracted concrete samples	Numeric data	Metric Generic Model/Structural entity
Corrosion Testing	Numeric data	Metric Generic Model/Structural entity
Survey of the reinforcements by georadar and pacometric test	Numeric data	Metric Generic Model/Structural entity
	Raster data	Surface area on the object
	3D geometry	Structural host object
Thermograms	Raster data	Surface area on the object
Environmental Temperature Monitoring	Numeric data	Environment

**Figure A1:**
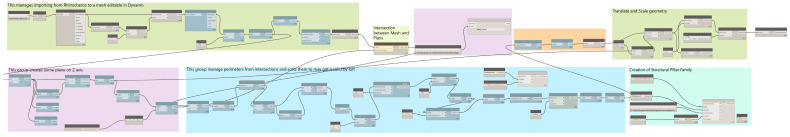
Diagram of the VPL script from Dynamo at high resolution.
